# The Antibacterial Activity of* Mass Galla Chinesis et Camelliae Fermentata* on* Helicobacter pylori* Infection

**DOI:** 10.1155/2018/1491732

**Published:** 2018-02-12

**Authors:** Jing Yu, Hui Ye, Jiang Li, Ning Li, Zong-ming Shi, Xue-zhi Zhang

**Affiliations:** ^1^Department of Integrated Traditional Chinese and Western Medicine, Peking University First Hospital, Beijing 100034, China; ^2^Department of Gastroenterology, Peking University First Hospital, Beijing 100034, China

## Abstract

*Mass Galla chinesis et camelliae Fermentata* (*Chinese gall leaven*, CGL) was investigated for activities against* Helicobacter pylori (H. pylori) *both* in vitro* and* in vivo*. The agar dilution method and time-kill curves, as* in vitro *assays and an* in vivo* study using a Kunming mice model, were performed. CGL demonstrated a strong anti-*Helicobacter pylori* activity* in vitro* with the minimal inhibitory concentrations (MICs) against multiple* H. pylori* strains of 0.5~8 mg/ml and the decreasing trend time-kill curves when increasing CGL concentrations.* H. pylori* eradication rates* in vivo *were evaluated based on rapid urease test (RUT) and histopathologic criteria. Results revealed that the eradication rates in the CGL groups were 40% (4/10) in the high dosage group, 33% (4/11) in the medium dosage group, and 18% (2/11) in the low dosage group, with the difference between the high dosage and* H. pylori *control groups being significant (*P* = 0.035). The* H. pylori* colonization scores could be reduced partly by CGL. These* in vivo* results demonstrated that CGL in a rationally high dosage might be the most effective.

## 1. Introduction

The discovery of* Helicobacter pylori (H. pylori)* in the 1980s caused a revolutionary change in the clinical treatment of gastritis and peptic ulcer disease [1]. Since then, the eradication of* H. pylori* has been recommended for* H. pylori*-associated diseases, and triple therapy, consisting of two antibiotics and one proton-pump inhibitor (PPI), became the standard treatment strategy and showed satisfying curative effect with the eradication rates > 90% [2]. However, the increasing rate of antibiotic resistance has resulted in the declined efficacy of triple therapy, which is no longer appropriate to be the first-line treatment in China due to the fact that the eradication rates could only reach 74.5%, currently [3]. Since the failure of standard triple therapy, alternative strategies, such as sequential, concomitant, and bismuth-containing quadruple therapies, have been applied, achieving promising results, and the updated Chinese guidance has recommended bismuth-containing quadruple therapy, composed of PPI, two antibiotics, and bismuth, as the primary clinical treatment, with the medication time extended to 10–14 days [4, 5]. However, the administration of long-term antibiotics produces more side effects and lowers patient compliance, and the occurrence of antibiotic resistance is still expected to increase. Therefore, searching for natural antibacterial agents to cure the pathogen is of concern. One hope as an alternative to antibacterial agents is traditional medicine that can effectively control this infection and eradicate* H. pylori* but with fewer side effects than antibiotics. As a source of greater chemical and bioactive diversity than synthetic drugs, traditional medicines offer a potential alternative for treatment methods. It is also important to identify effective and safe ones for* H. pylori *infection.

The fermentation processing of traditional Chinese medicines (TCMs) originated from the ancient brewing technology and had a long history in China. Fermented TCMs (FTCMs) had long been widely applied among folks for disease prevention and treatment [6].* Mass Galla chinesis et camelliae Fermentata* (Chinese gall leaven, CGL), as one of the FTCMs, has been used to treat digestive and some infectious diseases frequently. The fermentation preparation is with* Galla chinensis (Galla rhois)* as the raw material and* Thea viridis *as the fermentation substrate.* Galla chinensis *has been proven to be effective for several kinds of bacteria including* H. pylori* due to its active ingredient gallic acid.* Galla rhois* had long been shown to have the inhibitory effect on* H. pylori *growth and* H. pylori *urease in vitro [7], and its extract had demonstrated the effect to cure* H. pylori* infections and protect against* H. pylori*-induced pathology in a Mongolian gerbil model [8]. Gallic acid, as the active ingredient of* Galla chinensis*, also showed inhibitory effect on* H. pylori* strains and the effects were dependent on dose and contact time [9]. But, the further application of* Galla chinensis* is limited by the dyspepsia symptoms, which are caused by the excessive tannin ingredient [10]. The fermentation method can convert tannin to gallic acid, so the ingredients of CGL are more reasonable than* Galla chinensis* to human body. In this study, the anti-*Helicobacter *effects of CGL were investigated using* in vitro *time-kill curves and an* in vivo* study with a Kunming mice model.

## 2. Materials and Methods

### 2.1. Materials

*Helicobacter pylori* (NCTC 11637, 26695, 8 random clinical antibiotic-resistant strains and Sydney strain 1) were kindly donated by the Department of Gastroenterology, Peking University First Hospital. CGL (Hangzhou Jun Tong Pharmaceutical Co., Ltd., Hangzhou, China) was the drug tested. Lansoprazole, clarithromycin, and metronidazole (Beijing FreeMore Bioscience, Co., Ltd., Beijing, China) were used as the triple therapy control in animal experiments. Defibrinated sheep blood (Beijing Biotek Medical Device Ltd., Beijing, China), Columbia blood agar (OXOID Ltd., Basing Stoke, Hampshire, England), brain-heart infusion broth (OXOID Ltd., Basing Stoke, Hampshire, England), and drug-susceptibility test strips (*E*-test strip, AB BIODISK, Solna, Sweden) were used for the susceptibility test and* H. pylori* culture and collection. Brucella broth (BD Sparks, MD 21152, USA; 38800 Le Pont-de-Claix, France) and fetal calf serum (Beijing Solarbio Science & Technology Co., Ltd.) were prepared for the* H. pylori* liquid culture.

### 2.2. Preparation of Drugs

The drugs were ground into crude powders and then diluted in sterile water for preparation.

### 2.3. Assay of Inhibitory and Bactericidal Activities

Drug susceptibility was evaluated by the* E*-test.* H. pylori* (8 random clinical antibiotic-resistant strains; 3 × 10^8^ colony-forming units (CFU)/mL) were inoculated on Columbia blood agar containing 8% defibrinated sheep blood with* E*-test trip and cultured microaerobically (85% N_2_, 10% CO_2_, and 5% O_2_) at 37°C for 3 days. The minimal inhibitory concentration (MIC) value of metronidazole (MZ) and clarithromycin (CH) against* H. pylori *was read according to the specifications.* H. pylori* strains with MZ MIC > 8 mg/L or CH MIC > 1 mg/L were antibiotic-resistant. The experiments were performed in duplicate.

*H. pylori *growth-inhibitory activity of CGL was determined by the agar dilution MIC testing. The test was performed by adding serial dilutions of CGL to the Columbia blood agar plates with the final concentrations ranging from 8 to 0.25 mg/mL.* H. pylori *(NCTC11637, 26695 and 8 random clinical antibiotic-resistant strains; 3 × 10^8^ CFU/mL) was inoculated into the agar plates and cultured microaerobically at 37°C for 3 days. The minimal concentration of CGL that completely inhibited the growth of* H. pylori *was identified as the MIC. The non-drug agar was used as a negative control. All experiments were conducted in duplicate.

The bactericidal effect of CGL was examined by the time-kill curve methodology against the standard strain* H. pylori* NCTC11637. The strain was grown in the Columbia blood agar plates and incubated for 3 days at 37°C, followed by collecting the bacterial colonies in Brucella broth for preparation. The assays were performed using a shaking incubator set at 37.5°C, and four groups were set up, which were the 0.5x MIC CGL group, the 1.0x MIC CGL group, the 2.0x MIC CGL group, and a blank control group (in drug-free medium). The initial* H. pylori *inoculum was adjusted to 1 × 10^6^ CFU/mL; then 0.1 mL was extracted to be added to 90 mm plates, with the corresponding concentrations of CGL and Brucella broth containing fetal calf serum added previously. Each plate containing 10 mL of final volume was incubated under a microaerobic environment. Colony counts were determined at 0, 4, 8, and 24 h. At each time point, 0.5 mL of the mother liquid was removed from the plates and serial 10-fold dilutions were made in Brucella broth. 0.1 ml of the diluted solution was plated on Columbia blood agar plates and incubated at 37°C, and colonies were counted and averaged after 3 days, with bactericidal rate = (control colony count − medication colony count)/control colony count × 100%. In addition, the time-kill curves were drawn with a semilog plot. The assays were performed in duplicate.

### 2.4. Animal Experiments

Specific pathogen-free (SPF) male Kunming mice (22–28 g) were obtained from the Department of Laboratory Animal Science (Beijing, China; Certification Number SCXK 2011-0012) and kept in an isolated SPF barrier environment (room temperature, 23°C ± 1°C; humidity, 55% ± 5%; light/dark cycle, 12/12-hour), with free access to sterilized mouse feed and water. The experiment was performed on the basis of the Guide for Animal Experimentation of Peking University First Hospital and approved by the Experimental Animal Ethics Committee (Certification Number J201539). All the animals were allowed to acclimate for 1 week before the start of the experiment.

Sydney strain of* H. pylori* (strain SS1) was incubated on Columbia blood agar plates containing 8% defibrinated sheep blood at 37°C under a microaerobic environment for 2-3 days, and then the bacterial colonies were collected in brain-heart infusion broth for preparation. Mice (*n* = 65) were divided into two groups:* H. pylori*-uninfected group (*n* = 7) and* H. pylori*-infected group (*n* = 58). All the animals in the* H. pylori*-infected group were given 200 mg/kg cyclophosphamide by intraperitoneal injection and then inoculated six times at 2-day intervals with 0.4 mL of brain-heart infusion broth containing* H. pylori* (12 × 10^8^ CFU/mL) by intragastric gavage after fasting for 24 hours. Two weeks after* H. pylori* inoculation, one mouse in the* H. pylori*-uninfected group and three mice in the* H. pylori*-infected group were randomly selected and killed. The stomach samples were obtained and the* H. pylori* colonization was confirmed by both rapid urease test (RUT) and Warthin-starry (W-S) staining. After successfully establishing* H. pylori*-infected model, all surviving mice, 6 uninfected and 55 infected, were divided into the following six groups: blank control group (*H. pylori*-uninfected, *n* = 6),* H. pylori *control group (*H. pylori*-infected, *n* = 11), triple therapy (*H. pylori*-infected, *n* = 11), and CGL (high, medium, and low dosage,* H. pylori*-infected, *n* = 11 per group) groups. The triple therapy group was treated with a suspension of lansoprazole (12.33 mg/kg), clarithromycin (205.50 mg/kg), and metronidazole (164.40 mg/kg) daily for 1 week. The CGL groups were treated with high, medium, and low dosages of CGL daily for 4 weeks. Mice in the* H. pylori* group were treated with sterile water in the same volume. No intervention was given in the blank control group. The medium dosage that was equivalent to the clinical administration was 1.85 g/kg, while 3.7 g/kg was the high dosage and 0.925 g/kg was the low dosage. One mouse of the CGL-high dosage group died in the process of gavage administration due to the wrong operation. Four weeks after* in vivo* drug administration, all animals were killed to collect the stomachs, which were opened along the greater curvature and washed with 4°C PBS. Half of the gastric antrum was scraped to detect* H. pylori *colonization by RUT, and the rest was fixed in formalin and embedded in paraffin for histopathological observation. Eradication rates were determined by the results of RUT and W-S staining. The degree of* H. pylori* colonization was determined through histopathological observation and scored based on the visual analogue scales of the updated Sydney System [11]. The density of* H. pylori* was graded with the concepts of normal, mild, moderate, and marked, which were replaced with numbers “0,” “1,” “2,” and “3,” respectively, in this article.

### 2.5. Statistical Analysis

Fisher's Exact Test (SPSS 21.0; SPSS Inc., Chicago, IL, USA) was used for categorical data. Kruskal-Wallis ANOVA test was used for measurement data and a Dunn-Bonferroni test for post hoc comparisons. Data are expressed as mean ± SD, when appropriate. *P* < 0.05 was considered statistically significant.

## 3. Results

### 3.1. *In Vitro*

**Figure 1:**
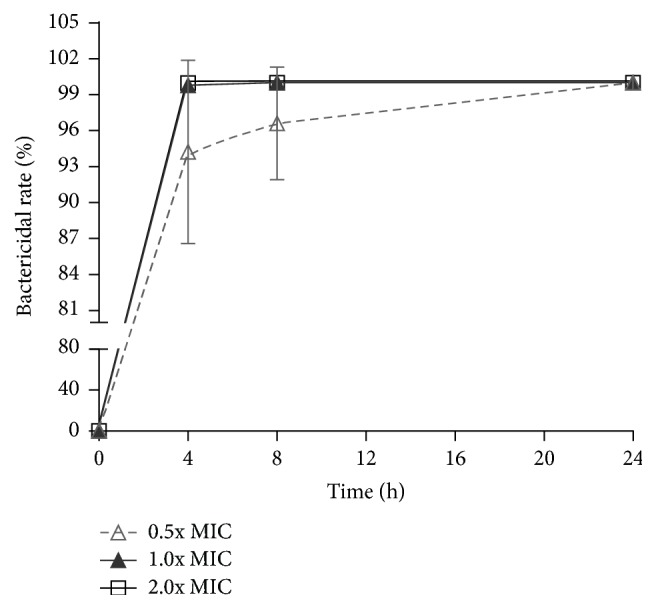
Bactericidal rate of CGL against* H. pylori* NCTC11637 at different concentrations. Data are represented as mean ± SD. MIC: minimal inhibitory concentration.

**Figure 2:**
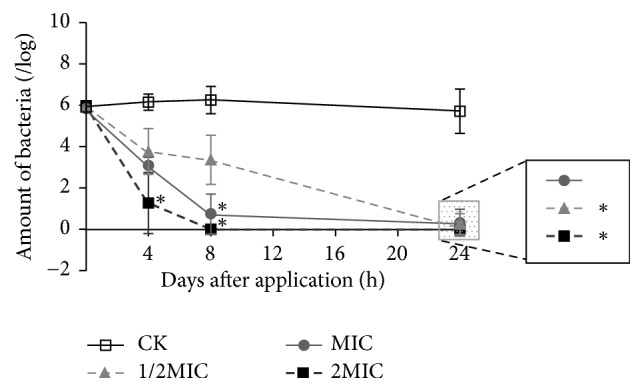
Time-kill curves of CGL against* H. pylori* NCTC11637 at different concentrations. Data are represented as mean ± SD. At each time point, *∗* represents the statistically significant difference between CK and other treatment groups at *P* < 0.05. CK: blank control group; MIC: minimal inhibitory concentration.

Antibiotic resistances of clinically isolated strains against MZ or CH were screened according to the* E-test*. The drug resistance situation against clinically common antibiotics is shown in Table 1.  Table 2 showed that CGL was effective against both antibiotic-resistant and sensitive strains with MICs of 0.5–8 mg/mL, while the MICs against antibiotic-resistant strains were lower than those against sensitive strains (0.5–2 versus 2–4 mg/mL), suggesting that CGL might be more useful against antibiotic-resistant strains.  Figure 1 showed the bactericidal activity of CGL against* H. pylori* NCTC11637 at different concentrations, and the effect increased gradually at each time point. The time-kill curves are shown in Figure 2. The results revealed that 0.5x MIC could achieve the complete elimination of* H. pylori* after 24 h and suggested a dose-effect relationship of CGL. Statistically significant differences could be observed between the blank control and the 2.0x MIC dosage groups at 4 h, the blank control and the 1.0x MIC/2.0x MIC dosage groups at 8 h, and the blank control and the 0.5x MIC/2.0x MIC dosage groups at 24 h.

### 3.2. *In Vivo*

**Figure 3:**
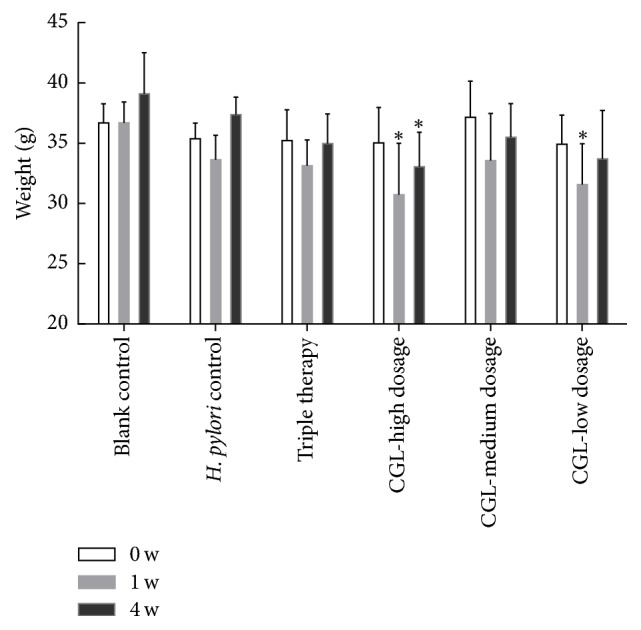
Effects of drug administration on body weight of mice. Data are represented as mean ± SD. At each time point, *∗* represents the statistically significant difference between blank control and other treatment groups at *P* < 0.05. Blank control group: *n* = 6;* Helicobacter pylori* group: *n* = 11; triple therapy group: *n* = 11; CGL-high dosage group: *n* = 10; CGL-medium dosage group: *n* = 11; CGL-low dosage group: *n* = 11. CGL*: Mass Galla chinesis et camelliae Fermentata*.

**Figure 4:**
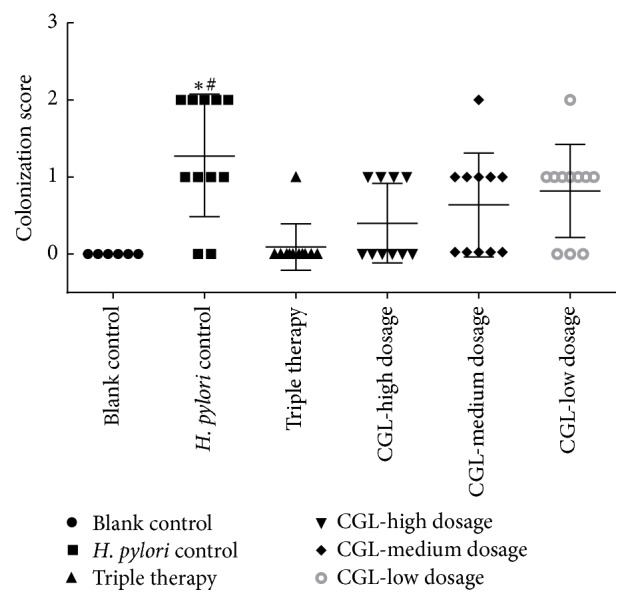
*Helicobacter pylori* colonization scores. Data are represented as mean ± SD. *∗* represents the statistically significant difference between blank control and other treatment groups at *P* < 0.05. # represents the statistically significant difference between triple therapy and other groups at *P* < 0.05. Blank control group: *n* = 6;* Helicobacter pylori* group: *n* = 11; triple therapy group: *n* = 11; CGL-high dosage group: *n* = 10; CGL-medium dosage group: *n* = 11; CGL-low dosage group: *n* = 11. CGL*: Mass Galla chinesis et camelliae Fermentata*.

**Figure 5:**
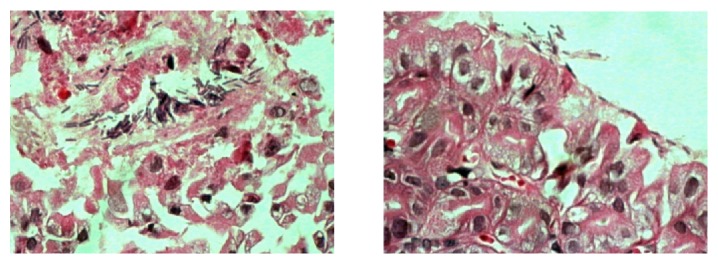
Histopathology of gastric mucosa. The successful colonization of* H. pylori* in gastric mucosa after* H. pylori* inoculation (magnification ×100).

The change in body weight during the treatment is shown in Figure 3. The mice had a weight loss in week 1 after* H. pylori* successfully colonized the stomach. After 4 weeks of treatment (CGL, 4 weeks; triple therapy, 1 week), the mice had a progressive weight gain, and CGL showed no obvious influence on the weight of mice. However, statistical differences were observed between the blank control and the high dosage groups in week 1 and week 4 and the blank control and the low dosage groups in week 1. The* H. pylori* eradication rates, which were determined by both RUT and histopathology, are presented in Table 3. The final eradication rates were 0 (0/11) in the* H. pylori *control group, 91% (10/11) in the triple therapy group, 40% (4/10) in the CGL-high dosage group, 33% (4/11) in the CGL-medium dosage group, and 18% (2/11) in the CGL-low dosage group. Significant differences existed between the* H. pylori *control and triple therapy/CGL-high dosage groups. Although the differences between the triple therapy and the CGL (high, medium, and low dosage) groups were significant (*P* = 0.024, *P* = 0.024, and *P* = 0.002), CGL showed a positive effect as a single drug. The* H. pylori *eradication rates of CGL (medium and low dosage) groups were greater than that of* H. pylori* control group; however, no significant differences were observed between them and thus a larger sample size will be needed. The* H. pylori* colonization scores are shown in Figure 4, with no bacteria colonization in the blank control group, “1” and “2” as the major degrees in the* H. pylori* control group, “0” in the triple therapy group, “0” and “1” in the CGL (high and medium dosage) groups, and “1” in the CGL-low dosage group. Statistically significant differences existed between* H. pylori* control and blank control/triple therapy groups. However, no significant differences were detected between the* H. pylori* control and the CGL (high, medium, and low dosage) groups. Similarly, there were also no statistically significant differences between the CGL groups and blank control/triple therapy groups.  Figure 5 shows the successful colonization of* H. pylori* in gastric mucosa after* H. pylori* inoculation.

## 4. Discussion

CGL has been utilized for the treatment of digestive system diseases with the effects of removing phlegm, helping produce saliva, and relieving thirst according to the traditional Chinese medicine (TCM) theory. Clinical observations indicated a significant improvement in clinical symptom alleviation in patients with gastric diseases concerning* H. pylori*. Nevertheless, current studies focus on its preparation technology. The anti-*H. pylori* effect remained unclear.

Our study confirmed the antibacterial activities of CGL both* in vivo* and* in vitro* by means of time-kill curves and* H. pylori*-infected mice model. Partly due to the limited data and the overly conservative statistical methods we adopted, significant differences were not detected between some groups, 1.0x MIC and blank control group in time-kill curve at the time point of 24 h, for example. These results strengthened the evidence for its clinical application. At present, CGL was applied to solve the complicated syndromes according to TCM theory and its satisfying safety with fewer side effects. This study expended its usage to the field of* H. pylori* infection treatment.

Despite the promising results, the eradication of CGL in vivo was not as ideal as that in vitro, possibly due to the gastric acid and mucus-bicarbonate barrier of the stomach. In CGL in* in vivo* experiment, the medium dosage was equivalent to the clinical administration, while the high dosage was double. However, the high dosage of CGL was excessively thick when converted to the animal dosage, and this may contribute to the relatively low weight of mice in the high dosage group. In addition, the body weight of mice had a slight decrease in week 1 after* H. pylori* successfully colonized the stomach, probably due to the initial, acute* H. pylori* infection phase. Considering the effect that excessively thick drug may cause and the weight gain that happened later, CGL showed no obvious influence on the weight of mice.

From the above, we learned that CGL was able to be an antibacterial agent. In addition, CGL also could be a health-promoting medicine. Through the fermentation, CGL is significantly different from* Galla chinensis *in its chemical component contents, accompanied with the increased gallic acid and reduced tannins [12]. Moderate tannins have anticancer effects [13], but foods rich in tannins are recognized as being of low nutritional value, taking into account the responsibilities for decreases in feed intake, growth rate, net metabolizable energy, feed efficiency, and protein digestibility [10]. Gallic acid has antimicrobial, anti-inflammation, and antioxidation effects [14–17]. A drug containing rich gallic acid and moderate tannins is the most reasonable to human body, and CGL meets the requirements. Thus, CGL not only can be an antibacterial agent but also has the potential to be a promising healthcare medicine. The results of the present study confirmed the anti-*H. pylori *activity of CGL, indicating that CGL at a rationally high dosage might be the most effective. However,* H. pylori* SS1 was chosen as the test strain in the* in vivo* study due to its stable colonization. Thus, the anti-*H. pylori *effects on drug-resistant isolates* in vivo* still need to be verified. Successfully establishing a stable drug-resistant animal model is essential for mimicking the human environment and further research determining the influence of the gastric environment on the anti-*H. pylori* activity of CGL would be helpful for improving its curative effect. Additionally, a combination of natural medicines has the prospect of becoming a better way to eradicate* H. pylori* since separate application may be slightly inferior to the antibiotics, and further research needs to be done.

## Figures and Tables

**Table 1:** Antibiotic resistance situation of *H. pylori* clinically isolated strains.

Strain number	MICs of antibiotics on *H. pylori* (*μ*g/ml)					
	AC	CH	TC	LE	MZ	MX
1	0.016	32^*∗*^	0.023	32^*∗*^	128^*∗*^	32^*∗*^
2	0.023	8^*∗*^	0.064	32^*∗*^	128^*∗*^	32^*∗*^
3	0.016	4^*∗*^	0.19	32^*∗*^	0.75	32^*∗*^
4	0.064	2^*∗*^	0.19	32^*∗*^	48^*∗*^	32^*∗*^
5	0.016	128^*∗*^	0.064	32^*∗*^	96^*∗*^	32^*∗*^
6	0.064	8^*∗*^	0.064	32^*∗*^	96^*∗*^	32^*∗*^
7	0.125	4^*∗*^	0.25	0.047	256^*∗*^	0.047
8	0.016	12^*∗*^	0.023	2^*∗*^	6	8^*∗*^

AC: amoxicillin; CH: clarithromycin; TC: tetracycline; LE: levofloxacin; MZ: metronidazole; MX: moxifloxacin.  ^*∗*^Resistant to the antibiotics.

**Table 2:** MICs of CGL against *H. pylori.*

Type	*H. pylori* strains	CGL concentration (mg/ml)					
		0.25	0.5	1	2	4	8
Drug-resistant	1	+	−	−	−	−	−
	2	+	−	−	−	−	−
	3	+	+	+	−	−	−
	4	+	−	−	−	−	−
	5	+	+	−	−	−	−
	6	+	+	+	−	−	−
	7	+	+	+	−	−	−
	8	+	+	+	−	−	−
Nonresistant	26695	+	+	+	+	+	−
	NCTC11637	+	+	+	+	−	−

+: existing colonies; −: no colony growth.

**Table 3:** *H. pylori* eradication rates.

Group	RUT (n)	W-S staining (n)	Eradication rate
Blank control	6	6	-
*H. pylori *control	0	2	0/11
Triple therapy	11	10	10/11^▲^
CGL-high dosage	4	6	4/10^▲#^
CGL-medium dosage	4	5	4/11^#^
CGL-low dosage	2	3	2/11^#^

^▲^ *P* < 0.05, versus *Helicobacter pylori* group; ^#^*P* < 0.05, versus triple therapy. Blank control group: *n* = 6; *Helicobacter pylori* group: *n* = 11; triple therapy group: *n* = 11; CGL-high dosage group: *n* = 10; CGL-medium dosage group: *n* = 11; CGL-low dosage group: *n* = 11. RUT: rapid urease test; CGL: *Mass Galla chinesis et camelliae Fermentata.*
